# Polyphyllin I induces mitophagic and apoptotic cell death in human breast cancer cells by increasing mitochondrial PINK1 levels

**DOI:** 10.18632/oncotarget.14413

**Published:** 2017-01-02

**Authors:** Guo-Bing Li, Ruo-Qiu Fu, Han-Ming Shen, Jing Zhou, Xiao-Ye Hu, Yan-Xia Liu, Yu-Nong Li, Hong-Wei Zhang, Xin Liu, Yan-Hao Zhang, Cheng Huang, Rong Zhang, Ning Gao

**Affiliations:** ^1^ Department of Pharmacognosy, College of Pharmacy, 3rd Military Medical University, Chongqing, China; ^2^ Department of Pharmacy, The Second Affiliated Hospital of Third Military Medical University, Chongqing, China; ^3^ Department of Physiology, Yong Loo Lin School of Medicine, National University of Singapore, Singapore, Republic of Singapore; ^4^ Drug Discovery Lab, School of Pharmacy, Shanghai University of Traditional Chinese Medicine, Shanghai, China

**Keywords:** polyphyllin I, DRP1, mitochondrial fission, mitophagy, PINK1

## Abstract

The molecular mechanisms underlying the anti-breast cancer effects of polyphyllin I, a natural compound extracted from *Paris polyphylla* rhizomes, are not fully understood. In the present study, we found that polyphyllin I induces mitochondrial translocation of DRP1 by dephosphorylating DRP1 at Ser637, leading to mitochondrial fission, cytochrome c release from mitochondria into the cytosol and, ultimately apoptosis. Polyphyllin I also increased the stabilization of full-length PINK1 at the mitochondrial surface, leading to the recruitment of PARK2, P62, ubiquitin, and LC3B-II to mitochondria and culminating in mitophagy. PINK1 knockdown markedly suppressed polyphyllin I-induced mitophagy and enhanced polyphyllin I-induced, DRP1-dependent mitochondrial fission and apoptosis. Furthermore, suppression of DRP1 by mdivi-1 or shRNA inhibited PINK1 knockdown/polyphyllin I-induced mitochondrial fragmentation and apoptosis, suggesting that PINK1 depletion leads to excessive fission and, subsequently, mitochondrial fragmentation. An *in vivo* study confirmed that polyphyllin I greatly inhibited tumor growth and induced apoptosis in MDA-MB-231 xenografts, and these effects were enhanced by PINK1 knockdown. These data describe the mechanism by which PINK1 contributes to polyphyllin I-induced mitophagy and apoptosis and suggest that polyphyllin I may be an effective drug for breast cancer treatment.

## INTRODUCTION

Autophagy is a cellular process in which organelles and proteins are sequestered into autophagic vesicles that are subsequently degraded through fusion with lysosomes [1]. Mitochondrial autophagy (hereafter referred to as mitophagy) is an important cellular pathway that facilitates the removal of damaged mitochondria [2, 3]. Mitochondria are very susceptible to reactive oxygen species (ROS)-induced damage [4]. Furthermore, mitochondrial damage depletes ATP and releases cytochrome *c* (Cyto C), which leads to the activation of caspases and, eventually, apoptosis [5]. The timely elimination of damaged mitochondria is therefore essential for maintaining the health of the cell. Mitophagy also plays an important role in the regulation of the tumor microenvironment and cancer cell death and survival, and studies of the molecular mechanisms underlying mitophagy in cancer will be crucial in developing novel therapies [6].

Mitophagy is regulated by the PINK1/PARK2 pathway. PARK2 is a RING domain-containing E3 ubiquitin ligase that can be activated through auto-ubiquitination [7]. When mitochondria are depolarized using mitochondrial uncoupling reagents such as CCCP (carbonyl cyanide m-chlorophenylhydrazone), PARK2 translocates to mitochondria and mediates mitochondrial degradation [8]. Furthermore, overexpression of PARK2 induces the degradation of depolarized mitochondria via mitophagy [9]. Because PARK2 also selectively binds only to damaged mitochondria, it might help to ensure the specificity of mitophagy [10].

PTEN-induced kinase 1 (PINK1), which contains a mitochondrial targeting sequence and is localized at the mitochondria [11]. PINK1 protects against neurotoxin-induced mitochondrial injury, while disease-associated PINK1 mutations or loss of PINK1 function result in ROS-mediated mitochondrial injury [12]. Only full-length PINK1 expression promotes autophagy or CCCP-mediated mitophagy [13]. Under stress conditions, mitochondrial membrane depolarization prevents mitochondrial uptake and processing of PINK1; the resulting accumulation of unprocessed PINK1 on the outer mitochondrial membrane recruits PARK2 and subsequently leads to elimination of damaged mitochondria via mitophagy [8]. PINK1 also regulates apoptosis and cell growth in breast cancer cells [14]. Because PINK1 regulates cancer cell survival, stress resistance, mitochondrial homeostasis, and cell cycle progression, it may serve as a therapeutic target or a predictive biomarker of response to treatment in cancer patients [15]. Inhibition of the fusion–fission cycle using the DRP1 inhibitor mdivi-1 prevents mitophagy, demonstrating the importance of mitochondrial fission in mitophagy [16]. DRP1-mediated mitochondrial fission induces LC3B lipidation and mitophagy, which requires PARK2 and PINK1 [17]. A recent study indicated that LC3B-II autophagosomes, which target mitochondrial membranes by interacting with C_18_-ceramide–LC3B-II, promote lethal mitophagy and suppress tumor growth [18]. An improved understanding of the molecular mechanisms by which DRP1-mediated mitochondrial fission affects mitophagy might help to identify potential drug targets for the treatment of various human cancers.

Polyphyllin I, a major steroidal saponin in extracts from *Paris polyphylla* rhizomes, has a wide range of biological activities against many types of cancers, including cervical, lung, ovarian, and gastric cancers, as well as osteosarcoma [19–24]. Polyphyllin I increases the sensitivity of hepatocellular carcinoma HepG2 cells to cisplatin [25]. Polyphyllin I also induces caspase-dependent apoptosis and activates autophagy via the PI3K/AKT/mTOR pathway in hepatocellular carcinoma HepG2 and SMCC7721 cells, and blockade of autophagy enhanced polyphyllin I-induced anti-proliferation effects [26]. Polyphyllin D (the same molecular structure as polyphyllin I) also induces apoptosis in human breast cancer MCF-7 and MDA-MB-231 cells via the mitochondrial pathway [27] and in drug-resistant HepG2 cells via mitochondrial fragmentation [28]. However, the exact mechanism by which polyphyllin I exerts anti-cancer effects in human breast cancer cells remains unclear.

In this study, we demonstrated for the first time that polyphyllin I induces apoptosis and mitophagy through DRP1-mediated mitochondrial fission. Notably, polyphyllin I treatment resulted in the accumulation of full-length PINK1 at the mitochondrial surface, which recruited PARK2 to the mitochondria and ultimately culminated in mitophagy. Polyphyllin I also induced mitochondrial translocation of DRP1 by dephosphorylating DRP1 at Ser637, which increased mitochondrial fission and apoptosis. shRNA-induced PINK1 knockdown combined with polyphyllin I treatment markedly decreased mitophagy and enhanced DRP1-dependent mitochondrial fission and apoptosis. Our study provides novel insight into the mitophagic and apoptotic effects of polyphyllin I and suggests that polyphyllin I may be a valuable chemotherapeutic agent for the clinical treatment of human breast cancer.

## RESULTS

### Polyphyllin I induces apoptosis through mitochondrial pathways

**Figure 1:**
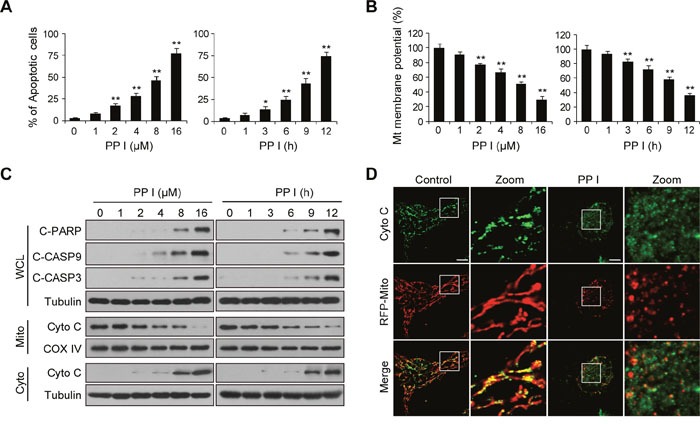
Polyphyllin I induces apoptosis via the mitochondrial pathway in MDA-MB-231 cells **A-B**. MDA-MB-231 cells were treated with various concentrations of polyphyllin I (PPI) for 9 h or with 8 μM PPI for different periods of time as indicated. Cells were stained using an Annexin V-FITC Apoptosis Detection Kit I or rhodamine 123, apoptosis and mitochondrial (Mt) membrane potential were detected with flow cytometry. Data are presented as mean ± SD (*P< 0.05 or **P< 0.01 vs. the control). **C**. Whole-cell lysates (WCL), mitochondrial (Mito), and cytosolic (Cyto) fractions were prepared and subjected to western blot analysis using antibodies against cleaved-PARP (C-PARP), cleaved-CASP9 (C-CASP9), cleaved-CASP3 (C-CASP3), and cytochrome c (Cyto C). Tubulin (whole-cell lysates) and COX IV (mitochondrial fraction) were used as the loading controls. **D**. MDA-MB-231 cells were transfected with RFP-mito plasmids and then exposed to PPI (8 μM) for 12 h. After immunostaining with Cyto C (Alexa Fluor 488, green), cells were examined by confocal microscopy. Scale bars: 10 μm.

We first evaluated the effects of polyphyllin I on apoptosis and mitochondrial membrane potential in breast cancer MDA-MB-231 cells using flow cytometry. Polyphyllin I treatment increased apoptosis and decreased mitochondrial potential in a dose- and time-dependent manner (Figure 1A and 1B). We then investigated whether polyphyllin I also induced apoptosis in MCF-7 breast cancer and Hs-578Bst human mammary stromal cells. Polyphyllin I induced apoptosis similarly in MCF-7 and MDA-MB-231 cells (Supplementary Figure 1). However, polyphyllin I induced apoptosis to a lesser degree in Hs-578Bst cells than in MDA-MB-231 and MCF-7 cells (Supplementary Figure 1). Western blot analysis revealed that polyphyllin I dose- and time-dependently increased cleavage/activation of CASP9 and CASP3, as well as increased PARP cleavage (Figure 1C). These events were also accompanied by significant increases in the release of Cyto C from the mitochondria into the cytosol (Figure 1C). An immunofluorescence assay also confirmed that Cyto C was released from mitochondria into the cytosol in response to polyphyllin I treatment (Figure 1D).

### Polyphyllin I induces mitochondrial fission and mitophagy in breast cancer cells

**Figure 2:**
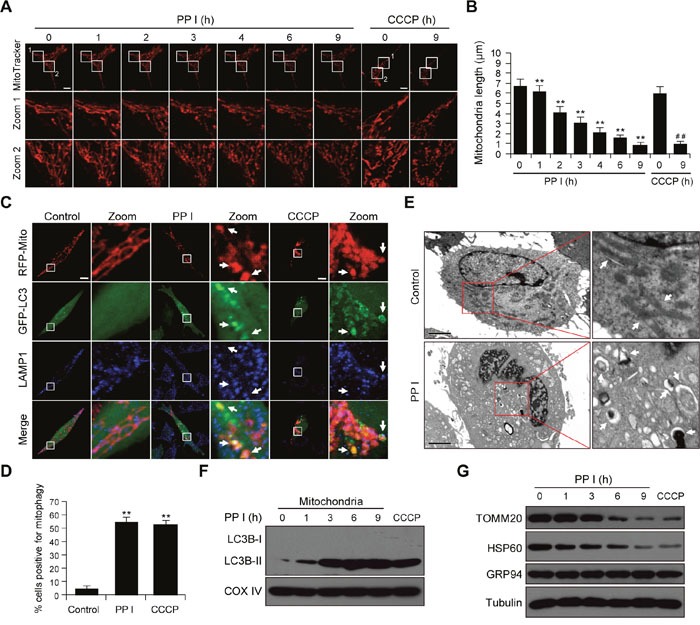
Polyphyllin I induces mitochondrial fission and mitophagy **A**. MDA-MB-231 cells were exposed to 8 μM PPI for different periods of time as indicated or treated with 20 μM CCCP (carbonyl cyanide m-chlorophenylhydrazone) for 9 h. Mitochondria were then stained using MitoTracker Red CMXRos and observed under a confocal microscope with a live cell imaging chamber. Scale bars: 10 μm. **B**. Average mitochondrial length was determined for 30 cells in each experiment; 3 independent experiments are included. Data are presented as mean ± SD (^*^P<0.01 vs. the control; ^##^*P*< 0.01 vs. the control). **C**. MDA-MB-231 cells were cotransfected with RFP-mito and GFP-LC3 and treated with 8 μM PPI or 20 μM CCCP for 9 h. After immunostaining with LAMP1 (Alexa Fluor 647, blue), cells were examined by confocal microscopy. Scale bars: 10 μm. **D**. The percentage of cells in which mitophagy occurred was determined using 30 cells from each experiment; 3 independent experiments are included. Cells with more than five RFP-Mito, LC3, and LAMP1 colocalization puncta were designated mitophagy-positive. Data are presented as mean ± SD (^*^P< 0.01 vs. the control). **E**. MDA-MB-231 cells were treated with 8 μM PPI for 9 h and imaged using a transmission electron microscope. Scale bars: 2 μm. Mitochondria and mitophagosomes were marked by white arrows. **F-G**. MDA-MB-231 cells were treated with 8 μM PPI for different periods of time as indicated or with 20 μM CCCP for 9 h. Mitochondrial fractions and whole-cell lysates were then prepared and subjected to western blot analysis.

Increasing evidence indicates that mitochondrial fission promotes the initiation of mitochondrial apoptosis [29]. We examined the effects of polyphyllin I on mitochondrial fission by staining MDA-MB-231 cells with the mitochondrion-selective probe Mitotracker Red CMXRos with CCCP as a positive control. Exposure of cells to polyphyllin I resulted in significantly increased mitochondrial fission and markedly decreased average mitochondria length in a time-dependent manner (Figure 2A and 2B). Similar results were observed in polyphyllin I-treated MCF-7 cells (Supplementary Figure 2A and 2B). Since mitochondrial fission isolates damaged mitochondria and promotes their elimination via mitophagy [30], we next examined whether polyphyllin I affects mitophagy. MDA-MB-231 cells transiently expressing RFP-Mito and GFP-LC3 were fluorescent-labeled with LAMP1 (Blue), and the colocalization of mitochondria, autophagosomes, and lysosomes was evaluated by fluorescent microscopy. GFP-LC3 colocalized with mitochondria and LAMP1 in polyphyllin I-treated MDA-MB-231 and MCF-7 cells (Figure 2C and 2D, Supplementary Figure 2C). Transmission electron microscopy (TEM) revealed high electron-density substances and abnormal mitochondria surrounded by double membranes, indicating the presence of mitophagosomes (Figure 2E). Western blot analysis revealed that polyphyllin I treatment increased LC3B-II levels in both mitochondrial fractions and whole-cell lysates in a time-dependent manner (Figure 2F, Supplementary Figures 2D and 3). Furthermore, polyphyllin I treatment decreased mitochondrial TOMM20 and HSP60 protein levels, but did not change levels of the ER protein GRP94, suggesting that polyphyllin I-induced autophagy mainly targets damaged mitochondria (Figure 2G). Taken together, these findings indicate that polyphyllin I induces mitochondrial fission and mitophagy in breast cancer cells.

To explore whether polyphyllin I induces autophagic flux or inhibits autophagic degradation, we treated cells with bafilomycin A_1_, a blocker of autophagosome and lysosome fusion. Immunofluorescence microscopy revealed that treatment with either polyphyllin I or bafilomycin A_1_ modestly increased numbers of GFP-LC3 puncta in MDA-MB-231 cells. Combined treatment with polyphyllin I and bafilomycin A_1_ further increased numbers of GFP-LC3 puncta (Supplementary Figure 4A and 4B). Western blots indicated that polyphyllin I treatment modestly increased LC3B-II accumulation, which was further increased by bafilomycin A_1_ treatment. Furthermore, the polyphyllin I-induced decrease in TOMM20 levels was largely reversed by bafilomycin A_1_ treatment (Supplementary Figure 4C). These findings suggest that polyphyllin I increased numbers of autophagosomes by increasing autophagosome formation rather than by inhibiting the fusion of autophagosomes with lysosomes.

### PINK1 and PARK2 are involved in polyphyllin I-induced mitophagy

**Figure 3:**
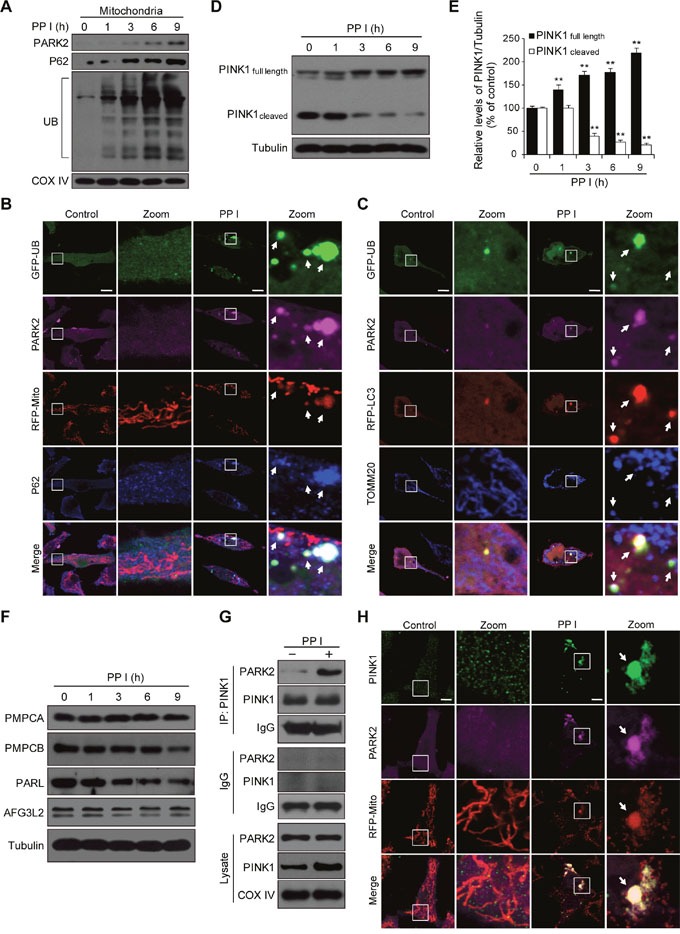
Polyphyllin I triggers PINK1/PARK2-dependent mitophagy **A**. MDA-MB-231 cells were treated with 8 μM PPI for different periods of time as indicated, and PARK2, P62, and ubiquitin (UB) levels in mitochondrial fractions were determined by western blot. **B**. Cells were cotransfected with GFP-UB and RFP-mito and treated with 8 μM PPI for 9 h, after which PARK2 (Alexa Fluor 405, pink) and P62 (Alexa Fluor 647, blue) immunostaining was detected using confocal microscopy. **C**. Cells cotransfected with GFP-UB and RFP-LC3 were treated with 8 μM PPI for 9 h, after which PARK2 (Alexa Fluor 405, pink) and TOMM20 (Alexa Fluor 647, blue) immunostaining was detected using confocal microscopy. Scale bars: 10 μm. **D-E**. MDA-MB-231 cells were treated with 8 μM PPI for different periods of time as indicated; whole-cell lysates were then separated on 8% SDS-PAGE gels and analyzed by western blot using the anti-PINK1 antibody. Relative full-length (∼63 kDa) and cleaved (∼52 kDa) PINK1 levels were quantified by densitometry and normalized to Tubulin. The results were expressed as a percentage of control, which was set at 100%. Data are presented as mean ± SD (^*^P< 0.01 vs. the control). **F**. Cells were treated with 8 μM PPI for different periods of time as indicated, and whole-cell lysates were then subjected to western blot analysis. **G**. Cells were treated with 8 μM PPI for 9 h, after which mitochondrial fractions were prepared and subjected to immunoprecipitation using anti-PINK1 antibody; associated PARK2 was detected using immunoblotting. **H**. RFP-mito-expressing MDA-MB-231 cells were treated with 8 μM PPI for 9 h, and PINK1 (Alexa Fluor 488, green) and PARK2 (Alexa Fluor 405, pink) immunostaining were evaluated using confocal microscopy. Scale bars: 10 μm.

PARK2 translocates to the mitochondrial surface and ubiquitinates numerous mitochondrial proteins, which in turn initiate mitophagy [8]. We therefore examined the effects of polyphyllin I on levels of PARK2, P62, and ubiquitin in mitochondria. Polyphyllin I treatment increased mitochondrial PARK2, P62, and ubiquitin levels (Figure 3A). An immunofluorescence assay revealed that PARK2 colocalized with mitochondria (RFP-Mito), P62, and ubiquitin in polyphyllin I-treated cells (Figure 3B). PARK2 also colocalized with LC3, the mitochondrial marker TOMM20, and ubiquitin in polyphyllin I-treated cells (Figure 3C). Thus, polyphyllin I treatment promotes recruitment of PARK2 to mitochondria, leading to the ubiquitination of mitochondrial proteins and colocalization with the UB-binding proteins P62 and LC3, and culminating in the degradation of mitochondria via mitophagy.

Since the recruitment of PARK2 to mitochondria is mainly dependent on PINK1, we next examined whether polyphyllin I affects PINK1 expression. Polyphyllin I treatment markedly increased levels of full-length PINK1 (∼63 kDa) and decreased levels of cleaved PINK1 (∼52 kDa) in a time-dependent manner (Figure 3D and 3E). Similar results were obtained in polyphyllin I-treated MCF-7 cells (Supplementary Figure 5). Next, we examined the effects of polyphyllin I treatment on the expression of the mitochondrial proteases peptidase mitochondrial processing alpha (PMPCA) and beta (PMPCB), presenilin-associated rhomboid-like protease (PARL), and AFG3L2 (a subunit of the m-AAA protease) [11, 31], all of which cleave PINK1. Polyphyllin I treatment decreased PMPCB and PARL levels, but did not alter PMPCA or AFG3L2 levels (Figure 3F). These results suggest that polyphyllin I induces the accumulation of full-length PINK1 by inhibiting the mitochondrial proteases PMPCB and PARL.

We then conducted an immunoprecipitation assay to investigate whether PARK2 binds to PINK1 in polyphyllin I-treated MDA-MB-231 cells. Interactions between PARK2 and PINK1 increased in polyphyllin I-treated cells (Figure 3G). Immunofluorescence microscopy revealed that PARK2 colocalized with PINK1 in the mitochondria after polyphyllin I treatment (Figure 3H). Taken together, these findings indicate that polyphyllin I induces the accumulation of full-length PINK1 at the mitochondrial surface by inhibiting the mitochondrial proteases PMPCB and PARL, in turn increasing PARK2 recruitment and mitophagy.

### Polyphyllin I induced, and PINK1 knockdown further increased, DRP1-dependent mitochondrial fission and apoptosis

**Figure 4:**
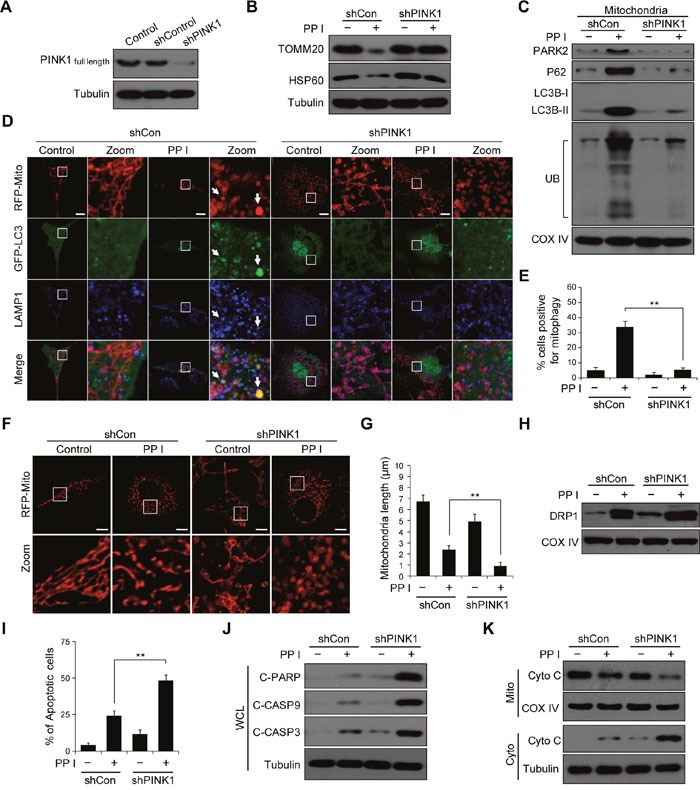
PINK1 knockdown combined with polyphyllin I treatment blocks mitophagy and increases mitochondrial fission and apoptosis **A**. MDA-MB-231 cells stably expressing non-target shRNA (shCon) or PINK1 shRNA (shPINK1) were lysed and analyzed by western blot. **B-C**. shCon and shPINK1 cells were treated with or without 8 μM PPI for 6 h; whole-cell lysates and mitochondrial fractions were then prepared and subjected to western blot analysis. **D-E**. shCon and shPINK1 cells were cotransfected with RFP-mito and GFP-LC3, and then treated with 8 μM PPI for 6 h. LAMP1 (Alexa Fluor 647, blue) immunostaining was then detected using confocal microscopy. Scale bars: 10 μm. The percentage of cells in which mitophagy occurred was determined using 30 cells from each experiment; 3 independent experiments are included. The cells with more than five RFP-Mito, LC3, and LAMP1 colocalization puncta were designated mitophagy-positive. Data are presented as mean ± SD (^*^P< 0.01 compared to shCon cells treated with PPI). **F-G**. shCon and shPINK1 cells were transfected with RFP-mito, and then treated with 8 μM PPI for 6 h. Mitochondria were observed using confocal microscopy. Scale bars: 10 μm. The average mitochondrial length was quantified as previously described. Data are presented as mean ± SD (^*^P< 0.01 compared to shCon cells treated with PPI). **H**. shCon and shPINK1 cells were treated with 8 μM PPI for 6 h, and DRP1 levels in mitochondrial fractions were determined by immunoblotting. **I**. Cells were treated with or without 8 μM PPI for 6 h, and apoptosis was then measured by flow cytometry. Data are presented as mean ± SD (^*^P< 0.01 compared to shCon cells treatment with PPI). **J-K**. Whole-cell lysates, mitochondrial (Mito), and cytosolic (Cyto) fractions were prepared and subjected to western blot analysis.

It has been shown that PINK1 silencing increases hydrogen peroxide-induced apoptosis in breast cancer cells [14]. Whether PINK1 knockdown could also sensitizes breast cancer cells to polyphyllin I, we used shRNA to stably knock down PINK1 expression (Figure 4A). PINK1 knockdown largely blocked polyphyllin I-induced reductions in mitochondrial TOMM20 and HSP60 protein levels (Figure 4B). Moreover, PINK1 knockdown decreased the polyphyllin I-induced increases in mitochondrial PARK2, P62, LC3B-II, and ubiquitin levels in both MDA-MB-231 and MCF-7 cells (Figure 4C, Supplementary Figure 6A-6C). An immunofluorescence assay revealed that PINK1 knockdown markedly reduced the polyphyllin I-induced colocalization of GFP-LC3 with mitochondria and LAMP1 (Figure 4D and 4E, Supplementary Figure 6D).

We then evaluated the effects of PINK1 knockdown on mitochondrial morphology. PINK1 knockdown increased polyphyllin I-induced mitochondrial fragmentation compared to shCon cells (Figure 4F and 4G, Supplementary Figure 7A and 7B). Because cytoplasmic DRP1 translocates to mitochondria and mediates mitochondrial fission [32], we next investigated whether PINK1 knockdown affects mitochondrial recruitment of DRP1 in response to polyphyllin I treatment. Western blots indicated that PINK1 knockdown markedly increased levels of DRP1 at mitochondria after polyphyllin I treatment compared to shCon cells (Figure 4H, Supplementary Figure 7C).

Because mitochondrial fragmentation is associated with increases in apoptosis and increased mitochondrial fission parallels the release of Cyto C from the mitochondria into the cytosol in apoptotic cells [33], we next examined the effects of PINK1 knockdown on apoptosis and Cyto C release in polyphyllin I-treated cells. PINK1 knockdown increased polyphyllin I-induced apoptosis, PARP cleavage, CASP9 and CASP3 activation, and Cyto C release (Figure 4I-4K, Supplementary Figure 7D and 7E). Taken together, these findings indicate that polyphyllin I induces mitophagy and DRP1-dependent mitochondrial fragmentation and apoptosis. Furthermore, PINK1 knockdown suppressed mitophagy and increased polyphyllin I-induced mitochondrial fragmentation and apoptosis.

### Suppression of DRP1 inhibits PINK1 knockdown-induced mitochondrial fragmentation and apoptosis after polyphyllin I treatment

**Figure 5:**
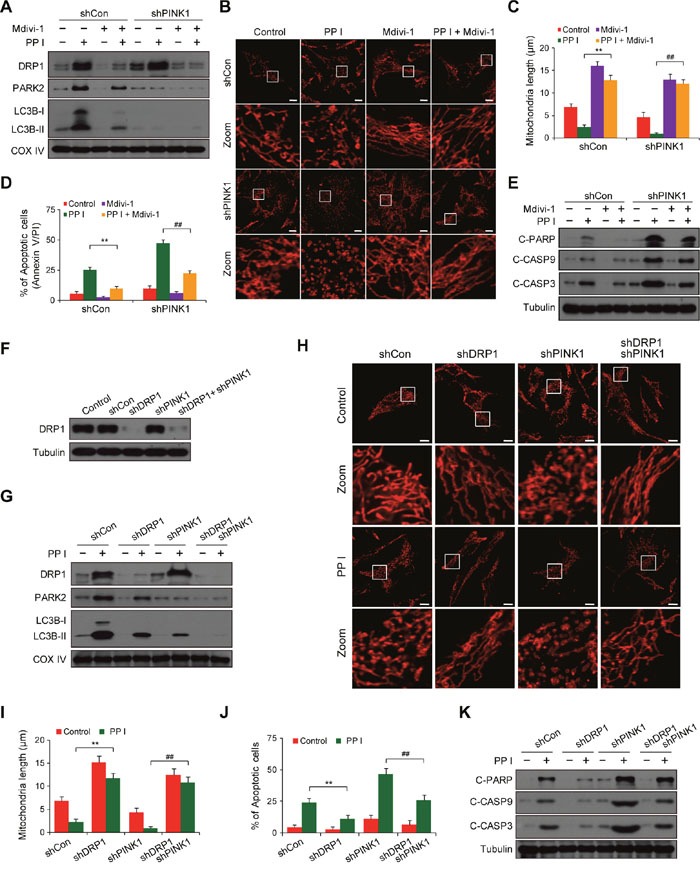
Suppression of DRP1 by mdivi-1 or shRNA blocks PINK1 depletion- and polyphyllin I-induced alterations in mitochondrial fission, mitophagy and apoptosis **A**. shCon and shPINK1 cells were pretreated with Mdivi-1 (50 μM) for 2 h, followed by treatment with 8 μM PPI for 6 h. Mitochondrial fractions (Mito) were prepared and subjected to western blot analysis. **B-C**. RFP-mito-expressing cells were treated as indicated in (A), and fluorescence images were evaluated by confocal microscopy. Scale bars: 10 μm. Average mitochondrial length was quantified as previously described. Data are presented as mean ± SD (^*^P< 0.01 compared to shCon cells treated with PPI, ^##^*P*< 0.01 compared to shPINK1 cells treated with PPI). **D-E**. Cells were treated as indicated in (A); apoptosis was then measured by flow cytometry, and whole-cell lysates were prepared and subjected to western blot analysis. Data are presented as mean ± SD (^*^P< 0.01 compared to shCon cells treated with PPI, ^##^*P*< 0.01 compared to shPINK1 cells treated with PPI). **F**. MDA-MB-231 cells were infected with shCon or shPINK1 and/or shDRP1 lentivirus; after selection with puromycin, cells were lysed and analyzed by western blot using the anti-DRP1 antibody. **G**. Cells were treated with 8 μM PPI for 6 h, and mitochondrial fractions were then prepared and subjected to western blot analysis. **H-I**. Cells were transfected with RFP-mito and then treated with 8 μM PPI for 6 h. Mitochondria were examined using confocal microscopy. Scale bars: 10 μm. Average mitochondrial length was quantified as previously described. Data are presented as mean ± SD (^*^P< 0.01 compared to shCon cells treated with PPI, ^##^*P*< 0.01 compared to shPINK1 cells treated with PPI). **J-K**. Apoptosis was measured by flow cytometry, and whole-cell lysates were prepared and subjected to western blot analysis. Data are presented as mean ± SD (^*^P< 0.01 compared to shCon cells treated with PPI, ^##^*P*< 0.01 compared to shPINK1 cells treated with PPI).

Because PINK1 knockdown increased mitochondrial fragmentation, we next investigated whether mitochondrial dynamics were perturbed by the loss of PINK1 function. Pretreatment with mdivi-1, a pharmacological inhibitor of DRP1, markedly inhibited the recruitment of DRP1 to mitochondria in both shCon and shPINK1 cells after polyphyllin I treatment (Figure 5A). Pretreatment with mdivi-1 also attenuated polyphyllin I-induced mitochondrial LC3B-II and PARK2 levels in both shCon and shPINK1 cells (Figure 5A). Moreover, mdivi-1 pretreatment blocked polyphyllin I-induced mitochondrial fragmentation in these cells (Figure 5B and 5C). Finally, mdivi-1 pretreatment attenuated polyphyllin I-induced apoptosis, PARP cleavage, and CASP9 and CASP3 activation in both shCon and shPINK1 cells (Figure 5D and 5E).

To exclude nonspecific effects of mdivi-1, we used a lentivirus shRNA approach to stably knock down DRP1 expression (Figure 5F). DRP1 knockdown markedly reduced polyphyllin I-induced increases in mitochondrial DRP1, PARK2, and LC3B-II levels in both shCon and shPINK1 cells (Figure 5G). DRP1 knockdown also markedly decreased polyphyllin I-induced mitochondrial fragmentation, apoptosis, PARP cleavage, and CASP9 and CASP3 activation in both shCon and shPINK1 cells (Figure 5H-5K). These results indicate that PINK1 knockdown in combination with polyphyllin I treatment increases mitochondrial fragmentation due to excessive fission.

### PINK1 knockdown promotes mitochondrial fragmentation and suppresses mitophagy by dephosphorylating DRP1 (Ser637)

**Figure 6:**
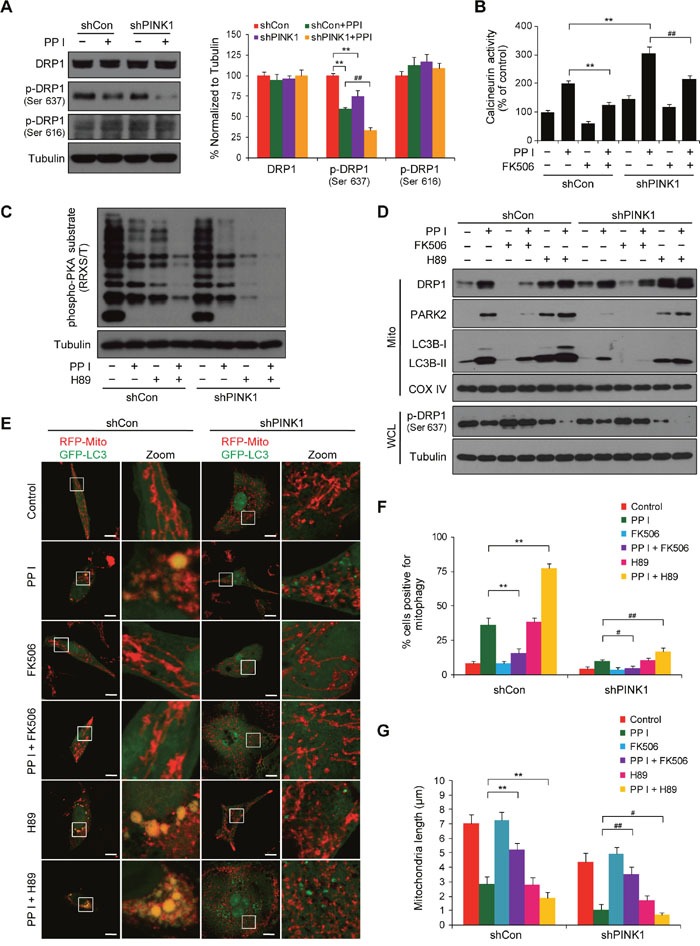
PINK1 depletion suppresses mitophagy and promotes mitochondrial fragmentation by dephosphorylating DRP1 at Ser637 **A**. shCon and shPINK1 cells were exposed to PPI (8 μM) for 6 h, after which whole-cell lysates were prepared and subjected to western blot analysis. Relative protein levels from 3 independent experiments were quantified by densitometry and normalized to Tubulin; the results are expressed as percentage of control level, which was set at 100%. Data are presented as mean ± SD (^*^P< 0.01 compared to shCon cells, ^##^*P*< 0.01 compared to shCon cells treated with PPI). **B**. Cells were pretreated with the calcineurin inhibitor FK506 (1 μM) for 2 h, followed by treatment with 8 μM PPI for 6 h; calcineurin activity was then measured as described in the Methods. Data are presented as mean ± SD (^*^P< 0.01 compared to shCon cells treated with PPI, ^##^*P*< 0.01 compared to shPINK1 cells treated with PPI). **C**. Cells were pretreated with the PKA inhibitor H89 (50 μM) for 2 h, followed by treatment with PPI (8 μM) for 6 h; whole-cell lysates were then prepared and subjected to western blot using the anti-phospho-PKA substrate (RRXS/T) antibody. **D**. Cells were pretreated with FK506 (1 μM) or H89 (50 μM) for 2 h and then exposed to PPI (8 μM) for 6 h, after which whole-cell lysates (WCL) and mitochondrial fractions (Mito) were prepared and subjected to western blot analysis. **E-G**. Cells were cotransfected with RFP-Mito and GFP-LC3 and treated as described in (D); fluorescence images were then evaluated using confocal microscopy. Scale bars: 10 μm. Percentages of mitophagy-positive cells and average mitochondrial length were measured as previously described. Data are presented as mean ± SD (^*^P< 0.01 compared to shCon cells treated with PPI, ^##^*P*< 0.05 or ^##^*P*< 0.01 compared to shPINK1 cells treated with PPI).

Dephosphorylation at Ser637 or phosphorylation at Ser616 both promote the translocation of DRP1 from the cytosol to mitochondria and increase mitochondrial fission [29]. We next examined the effects of PINK1 knockdown on DRP1 phosphorylation at Ser637 and Ser616 in polyphyllin I-treated cells. Western blot analysis revealed that PINK1 knockdown markedly increased polyphyllin I-induced dephosphorylation of DRP1 at Ser637 (Figure 6A). In contrast, polyphyllin I treatment alone or in combination with PINK1 knockdown did not alter DRP1 phosphorylation at Ser616 (Figure 6A).

DRP1 dephosphorylation at Ser637, which is due to the activity of the Ca^2+^-dependent phosphatase calcineurin (CaN), promotes mitochondrial fission, and protein kinase A (PKA)-mediated DRP1 phosphorylation at Ser637 causes mitochondrial elongation [34]. We therefore examined the effects of the CaN blocker FK506 and the PKA inhibitor H89 in cells with or without PINK1 knockdown. PINK1 knockdown markedly increased polyphyllin I-induced CaN activity, and pretreatment with FK506 largely blocked polyphyllin I-induced increases in CaN activity in both shCon and shPINK1 cells (Figure 6B). In addition, PINK1 knockdown markedly increased polyphyllin I-induced inhibition of PKA activity, and pretreatment with H89 increased polyphyllin I-induced inhibition of PKA activity in both shCon and shPINK1 cells (Figure 6C). Moreover, FK506 attenuated, while H89 enhanced, the polyphyllin I-mediated dephosphorylation of DRP1 at Ser637 and its translocation to mitochondria in both shCon and shPINK1 cells (Figure 6D).

Next, we investigated the effects of FK506 and H89 on polyphyllin I-induced changes in mitophagy and mitochondrial morphology in both shCon and shPINK1 cells. FK506 decreased, while H89 increased, polyphyllin I-induced increases in mitochondrial LC3B-II and PARK2 levels (Figure 6D). PINK1 knockdown markedly decreased polyphyllin I-induced increases in mitochondrial LC3B-II and PARK2 levels; the addition of FK506 further decreased, while H89 markedly increased, polyphyllin I-induced mitochondrial LC3B-II and PARK2 levels in shPINK1 cells (Figure 6D). Fluorescent microscopy revealed that FK506 decreased, while H89 increased, the colocalization of GFP-LC3 with mitochondria in polyphyllin I-treated shCon cells (Figure 6E and 6F). Compared to shCon cells, PINK1 knockdown decreased polyphyllin I-induced colocalization of GFP-LC3 with mitochondria. FK506 further decreased, while H89 increased, the polyphyllin I-induced colocalization of GFP-LC3 with mitochondria in shPINK1 cells (Figure 6E and 6F).

Moreover, FK506 blocked, and H89 increased, mitochondrial fragmentation in polyphyllin I-treated cells (Figure 6E-6G). Taken together, these findings suggest that knockdown of PINK1 enhances polyphyllin I-mediated dephosphorylation of DRP1 (Ser637) through promotion of CaN activity and suppression of PKA activity, leading to inhibition of mitophagy and induction of mitochondrial fragmentation.

### Polyphyllin I-induced suppression of tumor growth was enhanced by PINK1 knockdown in a MDA-MB-231 xenograft model

**Figure 7:**
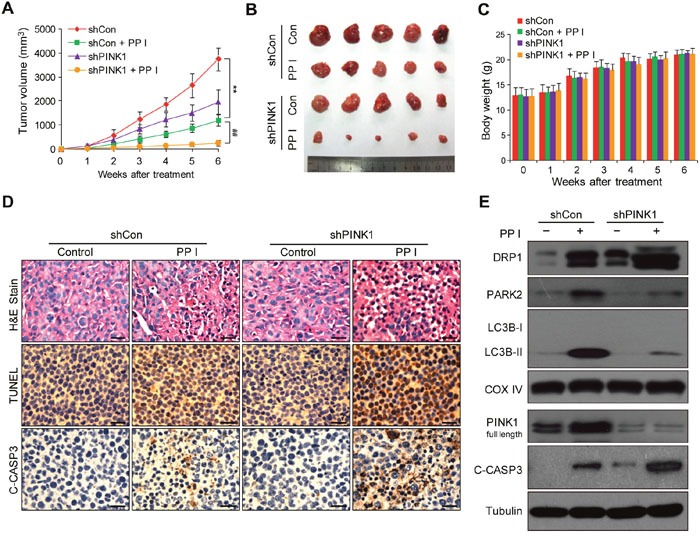
Polyphyllin I suppressed, and PINK1 knockdown further suppressed, tumor growth in a MDA-MB-231 xenograft model **A**. Tumor volumes were measured every week and differed at the end of the treatment period (^*^P < 0.01 compared to shCon cells treated with PPI, ^##^*P*< 0.01 compared to shPINK1 cells treated with PPI). **B**. Representative image of tumors from each group. **C**. Body weight changes in mice during the 6 weeks of PPI treatment. There were no differences in body weights between the PPI-treated shCon and vehicle-treated shCon groups or between the PPI-treated shPINK1 group and shCon groups. **D**. Representative tumor tissues were sectioned and subjected to H&E staining, TUNEL assay, and immunohistochemistry staining for C-CASP3. Scale bars: 50 μm. **E**. Representative tumor tissues from each group were prepared and subjected to western blot using anti-DRP1, -PARK2, -LC3B, -PINK1, and -C-CASP3 antibodies.

To determine whether our *in vitro* findings could be replicated *in vivo*, MDA-MB-231 cells stably expressing control shRNA or PINK1 shRNA were xenografted into immunodeficient nude mice, which then received intraperitoneal injections of either vehicle or polyphyllin I for 45 days. Tumor volumes were lower in mice with shPINK1 xenografts that were treated with polyphyllin I than in shCon xenograft mice treated with polyphyllin I (Figure 7A and 7B). Body weights did not differ among any of the groups of mice (Figure 7C), nor did other signs of potential toxicity, such as agitation, impaired movement and posture, and indigestion or diarrhea.

To determine whether PINK1 knockdown promotes polyphyllin I-induced apoptosis, hemotoxylin and eosin (H&E) staining, TdT-mediated dUTP nick-end labeling (TUNEL), and immunohistochemical analysis were performed. H&E staining of tumor sections from shPINK1 xenograft mice treated with polyphyllin I revealed changes in morphology, as indicated by signs of necrosis with infiltration of inflammatory cells and fibrosis, compared to shCon xenograft mice treated with polyphyllin I (Figure 7D). Additionally, polyphyllin I treatment dramatically increased numbers of TUNEL-positive cells and cleaved CASP3 immunoreactivity, which is indicative of apoptosis, in tumors from shPINK1 xenograft mice compared to shCon mice (Figure 7D).

We then used western blots to confirm that PINK1 knockdown enhanced polyphyllin I-induced apoptosis by promoting mitochondrial translocation of DRP1 and suppression of mitophagy. PINK1 knockdown dramatically increased DRP1 levels in mitochondrial fractions and decreased the mitochondrial translocation of PARK2 and LC3 compared to polyphyllin I-treated control mice (Figure 7E). Meanwhile, PINK1 knockdown enhanced polyphyllin I-induced CASP3 activation compared to polyphyllin I-treated control mice (Figure 7E). Taken together, these findings indicate that polyphyllin I inhibits MDA-MB-231 xenograft growth and that PINK1 knockdown enhances the antitumor activity of polyphyllin I by suppressing mitophagy and promoting DRP1-dependent mitochondrial fission and cell death.

## DISCUSSION

Mitochondria, which are commonly referred to as the power center of the cell, are involved in many biochemical functions, ranging from energy production to programmed cell death [5]. Damaged mitochondria undergo fission during apoptosis [35]; however, cells have developed a defense mechanism to protect against harm resulting from damaged mitochondria. This mechanism involves selective degradation of dysfunctional mitochondria by mitophagy [36]. Here, we show for the first time that polyphyllin I induced apoptosis and mitophagy in breast cancer cells. This polyphyllin I-induced mitophagy was characterized by the accumulation of LC3B-II in mitochondrial fractions, colocalization of GFP-LC3 with mitochondria and LAMP1, and downregulation of the mitochondrial proteins TOMM20 and HSP60. We demonstrated that mitophagy is an early response to polyphyllin I-induced mitochondrial injury that may help to efficiently eliminate unwanted or damaged mitochondria and re-establish mitochondrial homeostasis.

Mitophagy is regulated by the PINK1/PARK2 pathway. PINK1 and PARK2 cooperate to promote selective degradation of damaged mitochondria via mitophagy [37]. PINK1 recruits PARK2 from the cytoplasm to damaged mitochondria and subsequently promotes autophagy of the damaged mitochondria [13]. The chemotherapy drug doxorubicin increases PINK1 and PARK2 expression in the cerebral cortex [38]. In addition, overexpression of full-length PINK1 promotes mitophagy in the CCCP model [13]. In contrast, PINK1 knockdown suppresses mitophagy and promotes mitochondrial fragmentation and apoptosis [17]. Inhibition of autophagy enhanced polyphyllin I induced-apoptosis in human hepatocellular carcinoma cells [26]. The following results from this study strongly suggest that PINK1 plays an important role in the regulation of polyphyllin I-induced mitochondrial fission and mitophagy: (i) polyphyllin I induced the accumulation of full-length PINK1 by inhibiting the mitochondrial proteases PMPCB and PARL; (ii) PINK1 knockdown markedly decreased polyphyllin I-induced accumulation of mitochondrial LC3B-II and attenuated polyphyllin I-induced colocalization of GFP-LC3 with mitochondria and LAMP1; (iii) PINK1 knockdown largely blocked the polyphyllin I-induced reduction in levels of the mitochondrial proteins TOMM20 and HSP60; (iv) PINK1 knockdown increased polyphyllin I-induced mitochondrial fragmentation.

Mechanistically, we found that PINK1 contributes to polyphyllin I-induced mitophagy by recruiting PARK2 to the mitochondria. Polyphyllin I dramatically increased mitochondrial PARK2, P62, and ubiquitin levels in a time-dependent manner. In addition, PARK2 colocalized with mitochondria, P62, and ubiquitin, and with LC3, mitochondria, and ubiquitin in breast cancer cells after polyphyllin I treatment. Immunoprecipitation assays and immunofluorescence microscopy revealed that PARK2 and PINK1 interacted with each other and colocalized in the mitochondria in response to polyphyllin I treatment. Furthermore, PINK1 knockdown markedly decreased polyphyllin I-induced increases in mitochondrial PARK2, P62, LC3, and ubiquitin levels. These results suggest that polyphyllin I-induced mitophagy requires functional PINK1, which recruits PARK2 to the mitochondria and, in turn, ubiquitinates numerous mitochondrial proteins and recruits the ubiquitin- and LC3-binding adaptor protein P62 to mitochondria.

Mitochondria undergo frequent fission and fusion events that regulate their morphology. DRP1, a largely cytosolic member of the dynamin GTPase family, is a crucial regulator of mitochondrial fission [39], and disrupting mitochondrial fission with dominant-negative DRP1 prevents mitophagy [40]. In addition, PINK1 overexpression dramatically decreases mitochondrial DRP1 levels, while PINK1 knockdown markedly increases the translocation of DRP1 from the cytosol to mitochondria and promotes mitochondrial fragmentation [41, 42]. Several lines of evidence presented here indicate that PINK1 knockdown increases mitochondrial fragments was due to increased mitochondrial fragments generated rather than decreased the clearance by mitophagy. First, combined treatment with polyphyllin I and bafilomycin A_1_ revealed that polyphyllin I is an autophagic flux inducer. Second, PINK1 knockdown increased the polyphyllin I-induced translocation of DRP1 from the cytosol to mitochondria, but did not change levels of DRP1 in whole-cell lysates. Third, PINK1 knockdown dramatically increased polyphyllin I-induced mitochondrial fragmentation and mitochondrial apoptosis. Fourth, pretreatment with the DRP1 inhibitor mdivi-1 or DRP1 knockdown largely blocked polyphyllin I-induced mitochondrial fragmentation in PINK1-deficient cells.

Phosphorylation of different DRP1 serine residues regulates mitochondrial dynamics in various physiological and pathological processes, including mitosis, oxidative stress, nutrient starvation, and voltage-dependent calcium channel signaling [43]. Phosphorylation of the human brain DRP1 isoform (isoform 1) at serine 637 by protein kinase A inhibits mitochondrial division, while dephosphorylation of this residue by the calcium/calmodulin-dependent phosphatase calcineurin promotes mitochondrial fission [44]. Consistent with this report, our results strongly support that the loss of PINK1 activates dephosphorylation of DRP1 at serine 637, leading to mitochondrial fragmentation. First, PINK1 knockdown increased polyphyllin I-induced dephosphorylation of DRP1 at serine 637. Second, the calcineurin inhibitor FK506 inhibited, while the PKA inhibitor H89 dramatically enhanced, polyphyllin I-induced dephosphorylation and mitochondrial translocation of DRP1 in PINK1-deficient cells. Third, FK506 decreased and H89 dramatically increased polyphyllin I-induced mitochondrial fragmentation and mitophagy in PINK1-deficient cells. Taken together, these findings suggest that PINK1 knockdown suppresses mitophagy and promotes mitochondrial fragmentation by dephosphorylating DRP1 at serine 637.

PINK1 has important pro-survival, anti-apoptotic, and cytoprotective functions, suggesting that it may be a promising target for cancer therapies [15]. PINK1 deletion reduces, and PINK1 overexpression restores, cancer cell proliferation, colony formation, and invasiveness, indicating that PINK1 promotes cell cycle progression and acts as an oncogene [45]. Moreover, deletion of PINK1 sensitizes MCF-7 breast carcinoma cells, as well as other cell lines, to mitochondrial-dependent apoptotic death stimuli [14, 46]. Consistent with these reports, our results revealed that PINK1 knockdown in combination with polyphyllin I treatment suppressed mitophagy and promoted DRP1-dependent mitochondrial fission and apoptosis, suggesting that PINK1 might be an important therapeutic target of polyphyllin 1-based breast cancer treatments.

**Figure 8:**
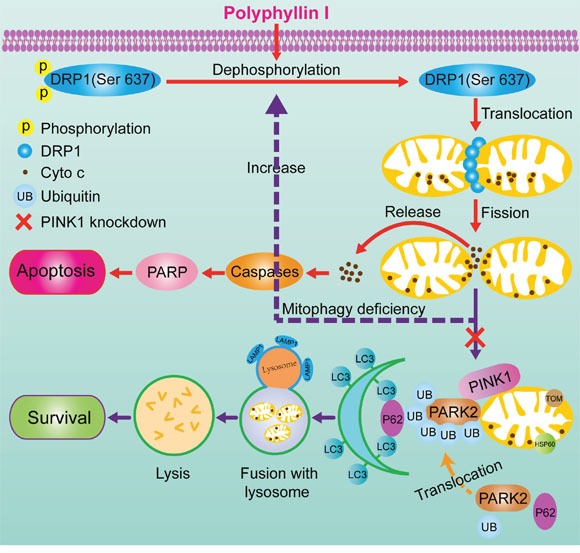
A proposed model for polyphyllin I-induced mitophagic and apoptotic cell death in human breast cancer cells Polyphyllin I induced mitochondrial translocation of DRP1 by dephosphorylating DRP1 at Ser637, which lead to mitochondrial fission and cytochrome c release from the mitochondria into the cytosol, in turn activating caspases and promoting apoptosis. Meanwhile, polyphyllin I also increased stabilization of full-length PINK1 at the mitochondrial surface, leading to recruitment of PARK2, P62, ubiquitin, and LC3B-II to the mitochondria and culminating in mitophagy. Moreover, PINK1 knockdown markedly suppressed mitophagy and enhanced polyphyllin I-induced, DRP1-dependent mitochondrial fission and apoptosis.

In conclusion, we provide evidence that mitophagy promotes cellular survival during the polyphyllin I-induced preapoptotic lag phase, leading to delayed mitochondrial apoptosis. Polyphyllin I-induced mitophagy suppressed mitochondrial apoptosis, while PINK1 knockdown-induced inhibition of mitophagy accelerated DRP1-dependent mitochondrial fragmentation, leading to mitochondrial apoptosis in response to polyphyllin I treatment (Figure 8). Our study describes a mechanism by which PINK1 mediates the complex balance between polyphyllin I-induced mitophagy and mitochondrial fission-mediated apoptosis in breast cancer cells. Our findings suggest that polyphyllin I might be an effective novel agent for breast cancer treatment.

## MATERIALS AND METHODS

### Reagents and antibodies

Polyphyllin I (A0386) was purchased from Must bio-technology (Chengdu, China). Mitotracker Red CMXRos (M7512) was purchased from Molecular Probes. Mdivi-1 (S7162), FK506 (S5003), and H89 (S1582) were purchased from Selleck Chemicals. The following antibodies were used: Cleaved-CASP9 (9505), Cleaved-CASP3 (9661), COX IV (4850), Ubiquitin (3936), LAMP1 (9091), P62 (5114), HSP60 (12165), phospho-PKA Substrate (9624), phospho-DRP1 Ser616 (3455), and phospho-DRP1 Ser637 (4867) were from Cell Signaling Technology; Tubulin (sc-23948), Cytochrome *c* (sc-13156), PARK2 (sc-32282), PMPCA (sc-390471), PMPCB (sc-160672), PARL (sc-133884), AFG3L2 (sc-84687), and TOMM20 (sc-11415) were from Santa Cruz Biotechnology; DRP1 (611113) was from BD Biosciences; PINK1 (BC100-494) was from Novus; LC3B (L7543) was from Sigma; Cleaved-PARP (ab32071) was from Abcam.

### Cell culture

MDA-MB-231 (HTB-26) and MCF-7 (HTB-22) cells were obtained from American Type Culture Collection and cultured in Dulbecco's modified Eagle medium (DMEM) supplemented with 10% FBS. 293FT cells (Invitrogen, R700-07) were maintained in DMEM supplemented with 10% FBS, 0.1 mM non-essential amino acids (Gibco, 11140), 4 mM L-glutamine (Gibco, 25030-081), 1% penicillin/streptomycin, and 0.5 mg/mL G418 (Sigma, A1720).

### Apoptosis and mitochondrial membrane potential assay

Apoptosis was evaluated using the Annexin V-FITC Apoptosis Detection Kit I (BD Biosciences, 556547) according to the manufacturer's instructions. The mitochondrial membrane potential (MMP) was monitored using Rhodamine 123 (Molecular Probes, R-22420). Briefly, cells were incubated with 5 μM Rhodamine 123 for 10 min at 37°C, washed with PBS, and subsequently analyzed by flow cytometry (FACScan, Becton Dickinson).

### Western blots and immunoprecipitation

Mitochondrial and cytosolic fractions were isolated using the Mitochondria Isolation Kit for Cultured Cells (Pierce, 89874) according to the manufacturer's protocols. For total protein extraction, cells were lysed in RIPA buffer (Beyotime, China, P0013B). Total protein concentration was detected using the Enhanced BCA Protein Assay Reagent (Beyotime, China, P0010), and equal amounts of each sample were boiled with 1× SDS-PAGE sample buffer for 10 min, separated on 8% - 14% SDS-PAGE gels, and transferred to PVDF membranes (Bio-Rad, 162-0177). After blocking with 5% nonfat dried milk, the blots were incubated with the appropriate antibodies and bands were visualized using the enhanced chemiluminescence kit (Bio-Rad, 170-5061). For immunoprecipitation, equal quantities of proteins were incubated with PINK1 antibody at 4°C for 12 h followed by incubation with protein A/G agarose beads (Santa Cruz Biotechnology, sc-2003) for 3 h, after which immune complexes were collected by centrifugation. After washing 5 times in phosphate-buffered saline (PBS), samples were subjected to western blot analysis. Densitometric analysis of the blots was performed using Quantity One software (Bio-Rad, Germany).

### Plasmids and establishment of stable cell lines

Human PTEN-induced putative kinase 1 shRNA (shPINK1) plasmid (SHCLND-NM_032409), DRP1 shRNA (shDRP1) plasmid (SHCLND-NM_ 012062) and pLKO.1-puro Non-Target shRNA Control (shCon) Plasmid (SHC016-1EA) were purchased from Sigma. 293FT cells were co-transfected with lentiviral packaging vectors pLP1, pLP2, and pLP/VSVG (Invitrogen, K4975) along with shPINK1, shDRP1, or shCon plasmid using Lipofectamine 3000 (Invitrogen, L3000015) according to the manufacturer's protocols. 48 h later, supernatant containing the lentivirus was harvested and infected MDA-MB-231 cells. Cells were subsequently selected with 5 μg/mL puromycin (Sigma, P9620) to establish stable cell lines.

### Transmission electron microscopy assay

Cells were fixed in glutaraldehyde (2.5% in PBS) at 4°C for 24 h, then fixed in 2% osmium tetroxide at 4°C for 2 h, dehydrated with ethanol, and embedded in Epon. Ultra-thin sections were prepared using a microtome (UC7, Leica, Germany) and stained with uranyl acetate and lead citrate. Sections were examined under a Tecnai 10 transmission electron microscope (Philips, Netherlands).

### Immunofluorescence

Cells were seeded on coverslips and cultured in 24-well plates for 24 h, followed by transfection with GFP-UB (11928) and RFP-LC3 (21075) (Addgene, USA) or RFP-mito and GFP-LC3 (GeneChem, Biotechnology, Shanghai, China) plasmids using Lipofectamine 3000 (Invitrogen, L3000015) according to the manufacturer's instructions. 48 h after transfection, cells were treated with different drugs for the indicated amounts of time, and then fixed with 4% formaldehyde for 15 min, permeabilized with 0.1% Triton X-100 for 10 min, and blocked with 10% FBS for 30 min. Cells were incubated with various primary antibodies at 4°C overnight, followed by incubation with the following secondary antibodies at 37°C for 1 h, as appropriate: Alexa Fluor 488 goat anti-mouse (Molecular Probes, A11001), Alexa Fluor 405 goat anti-mouse (Molecular Probes, A-31553), or Alexa Fluor 647 donkey anti-rabbit (Molecular Probes, A31573). Cells were visualized using a laser-scanning confocal microscope (LSM780NLO, Zeiss, Germany) or a confocal microscope with a live cell imaging chamber (DMI 6000B, Leica, Germany). Mitochondrial length and percentage of cells in which mitophagy occurred were determined blindly using randomized filenames (Filename randomizer, CodeUnit, Craig Lotter) and Zeiss LSM Image Examiner software.

### Calcineurin activity assay

The calcineurin activity assay was performed using the Calcineurin Cellular Activity Assay Kit (Calbiochem, 207007) according to the manufacturer's instructions. Briefly, cell extracts were desalted using a resin chromatography column to remove excess phosphate and nucleotides, and 5 μL of extract was then added to each well of a 96-well plate containing 10 μL phosphopeptide substrate (1.64 mg/mL) with or without 25 μL 2× EGTA buffer. The plate was incubated at 30°C for 30 min, followed by an additional 30 min incubation with 100 μL GREEN™ reagent at room temperature. Absorbance was read at 620 nm using a microplate reader (Bio-Rad, Germany). Calcineurin activity was calculated according to the formula (A_Calcineurin_ = A_Total_- A_EGTA_) and normalized to the control group.

### Animal experiments

All animal studies were approved by the Third Military Medical University Institutional Animal Care and Use Committee. Female nude mice (5 weeks old) were purchased from Vital River Laboratories (VRL, Beijing, China). Cells stably expressing shCon or shPINK1 (1×10^7^) were mixed with Matrigel (1:1, BD Biosciences, 354234) and injected subcutaneously into the mammary fat pads of nude mice. Mice were randomized into four groups (n = 40, 10 mice per group). Polyphyllin I (5 mg/kg) or an equal volume of vehicle was administered daily by intraperitoneal injection starting 7 days after tumor inoculation. Tumor growth and body weights were measured every week, and tumor volume was calculated as (length ×width^2^)/2. After 45 days of treatment, mice were euthanized by cervical dislocation, and tumor tissues were harvested and fixed in formalin or frozen at -80°C. Histological, TUNEL, and immunohistochemical analysis were performed as previously described [47]. Representative tumor tissues from each group were lysed and subjected to western blot analysis.

### Statistical analysis

Data are presented as mean ± SD. Comparisons between groups were performed using one-way ANOVAs. *P* < 0.05 was considered statistically significant.

## SUPPLEMENTARY FIGURES



## References

[1] Levine B, Kroemer G (2008). Autophagy in the pathogenesis of disease. Cell.

[2] Zhu J, Wang KZ, Chu CT (2013). After the banquet: mitochondrial biogenesis, mitophagy, and cell survival. Autophagy.

[3] Wang K, Klionsky DJ (2011). Mitochondria removal by autophagy. Autophagy.

[4] Venditti P, Di Stefano L, Di Meo S (2013). Mitochondrial metabolism of reactive oxygen species. Mitochondrion.

[5] Tait SW, Green DR (2010). Mitochondria and cell death: outer membrane permeabilization and beyond. Nat Rev Mol Cell Biol.

[6] Lu H, Li G, Liu L, Feng L, Wang X, Jin H (2013). Regulation and function of mitophagy in development and cancer. Autophagy.

[7] Beasley SA, Hristova VA, Shaw GS (2007). Structure of the Parkin in-between-ring domain provides insights for E3-ligase dysfunction in autosomal recessive Parkinson's disease. Proc Natl Acad Sci U S A.

[8] Narendra D, Tanaka A, Suen DF, Youle RJ (2008). Parkin is recruited selectively to impaired mitochondria and promotes their autophagy. J Cell Biol.

[9] Vives-Bauza C, Zhou C, Huang Y, Cui M, de Vries RL, Kim J, May J, Tocilescu MA, Liu W, Ko HS, Magrane J, Moore DJ, Dawson VL (2010). PINK1-dependent recruitment of Parkin to mitochondria in mitophagy. Proc Natl Acad Sci U S A.

[10] Cai Q, Zakaria HM, Simone A, Sheng ZH (2012). Spatial parkin translocation and degradation of damaged mitochondria via mitophagy in live cortical neurons. Curr Biol.

[11] Jin SM, Lazarou M, Wang C, Kane LA, Narendra DP, Youle RJ (2010). Mitochondrial membrane potential regulates PINK1 import and proteolytic destabilization by PARL. The Journal of cell biology.

[12] Gautier CA, Kitada T, Shen J (2008). Loss of PINK1 causes mitochondrial functional defects and increased sensitivity to oxidative stress. Proc Natl Acad Sci U S A.

[13] Matsuda N, Sato S, Shiba K, Okatsu K, Saisho K, Gautier CA, Sou YS, Saiki S, Kawajiri S, Sato F, Kimura M, Komatsu M, Hattori N (2010). PINK1 stabilized by mitochondrial depolarization recruits Parkin to damaged mitochondria and activates latent Parkin for mitophagy. J Cell Biol.

[14] Berthier A, Navarro S, Jimenez-Sainz J, Rogla I, Ripoll F, Cervera J, Pulido R (2011). PINK1 displays tissue-specific subcellular location and regulates apoptosis and cell growth in breast cancer cells. Hum Pathol.

[15] O'Flanagan CH, O'Neill C (2014). PINK1 signalling in cancer biology. Biochim Biophys Acta.

[16] Dagda RK, Cherra SJ, Kulich SM, Tandon A, Park D, Chu CT (2009). Loss of PINK1 function promotes mitophagy through effects on oxidative stress and mitochondrial fission. J Biol Chem.

[17] Lutz AK, Exner N, Fett ME, Schlehe JS, Kloos K, Lammermann K, Brunner B, Kurz-Drexler A, Vogel F, Reichert AS, Bouman L, Vogt-Weisenhorn D, Wurst W (2009). Loss of parkin or PINK1 function increases Drp1-dependent mitochondrial fragmentation. J Biol Chem.

[18] Sentelle RD, Senkal CE, Jiang W, Ponnusamy S, Gencer S, Selvam SP, Ramshesh VK, Peterson YK, Lemasters JJ, Szulc ZM, Bielawski J, Ogretmen B (2012). Ceramide targets autophagosomes to mitochondria and induces lethal mitophagy. Nat Chem Biol.

[19] Cheng H, Su JJ, Hou HJ, Li QL (2013). Effect and mechanism of polyphyllin I on human cervical cancer cell HeLa in vitro. [Article in Chinese]. Zhong Yao Cai.

[20] Chen Y, Zhu J, Zhang W (2014). Antitumor effect of traditional Chinese herbal medicines against lung cancer. Anticancer Drugs.

[21] Gu L, Feng J, Xu H, Luo M, Su D (2013). Polyphyllin I inhibits proliferation and metastasis of ovarian cancer cell line HO-8910PM in vitro. J Tradit Chin Med.

[22] Kong M, Fan J, Dong A, Cheng H, Xu R (2010). Effects of polyphyllin I on growth inhibition of human non-small lung cancer cells and in xenograft. Acta biochimica et biophysica Sinica.

[23] Chang J, Wang H, Wang X, Zhao Y, Zhao D, Wang C, Li Y, Yang Z, Lu S, Zeng Q, Zimmerman J, Shi Q, Wang Y (2015). Molecular mechanisms of Polyphyllin I-induced apoptosis and reversal of the epithelial-mesenchymal transition in human osteosarcoma cells. J Ethnopharmacol.

[24] Yue G, Wei J, Qian X, Yu L, Zou Z, Guan W, Wang H, Shen J, Liu B (2013). Synergistic anticancer effects of polyphyllin I and evodiamine on freshly-removed human gastric tumors. PLoS One.

[25] Han W, Hou G, Liu L (2015). Polyphyllin I (PPI) increased the sensitivity of hepatocellular carcinoma HepG2 cells to chemotherapy. Int J Clin Exp Med.

[26] Shi YM, Yang L, Geng YD, Zhang C, Kong LY (2015). Polyphyllin I induced-apoptosis is enhanced by inhibition of autophagy in human hepatocellular carcinoma cells. Phytomedicine.

[27] Lee MS, Yuet-Wa JC, Kong SK, Yu B, Eng-Choon VO, Nai-Ching HW, Chung-Wai TM, Fung KP (2005). Effects of polyphyllin D, a steroidal saponin in Paris polyphylla, in growth inhibition of human breast cancer cells and in xenograft. Cancer Biol Ther.

[28] Ong RC, Lei J, Lee RK, Cheung JY, Fung KP, Lin C, Ho HP, Yu B, Li M, Kong SK (2008). Polyphyllin D induces mitochondrial fragmentation and acts directly on the mitochondria to induce apoptosis in drug-resistant HepG2 cells. Cancer Lett.

[29] Sheridan C, Martin SJ (2010). Mitochondrial fission/fusion dynamics and apoptosis. Mitochondrion.

[30] Twig G, Elorza A, Molina AJ, Mohamed H, Wikstrom JD, Walzer G, Stiles L, Haigh SE, Katz S, Las G, Alroy J, Wu M, Py BF (2008). Fission and selective fusion govern mitochondrial segregation and elimination by autophagy. EMBO J.

[31] de Vries RL, Przedborski S (2013). Mitophagy and Parkinson's disease: be eaten to stay healthy. Mol Cell Neurosci.

[32] Youle RJ, van der Bliek AM (2012). Mitochondrial fission, fusion, and stress. Science.

[33] Chan DC (2012). Fusion and fission: interlinked processes critical for mitochondrial health. Annu Rev Genet.

[34] Otera H, Mihara K (2011). Molecular mechanisms and physiologic functions of mitochondrial dynamics. J Biochem.

[35] Li MX, Dewson G (2015). Mitochondria and apoptosis: emerging concepts. F1000Prime Rep.

[36] Jourdain A, Martinou JC (2009). Mitochondrial outer-membrane permeabilization and remodelling in apoptosis. Int J Biochem Cell Biol.

[37] Melser S, Lavie J, Benard G (2015). Mitochondrial degradation and energy metabolism. Biochim Biophys Acta.

[38] Marques-Aleixo I, Santos-Alves E, Balca MM, Moreira PI, Oliveira PJ, Magalhaes J, Ascensao A (2016). Physical exercise mitigates doxorubicin-induced brain cortex and cerebellum mitochondrial alterations and cellular quality control signaling. Mitochondrion.

[39] Bertolin G, Ferrando-Miguel R, Jacoupy M, Traver S, Grenier K, Greene AW, Dauphin A, Waharte F, Bayot A, Salamero J, Lombes A, Bulteau A-L, Fon EA (2013). The TOMM machinery is a molecular switch in PINK1 and PARK2/PARKIN-dependent mitochondrial clearance. Autophagy.

[40] Geisler S, Holmstrom KM, Skujat D, Fiesel FC, Rothfuss OC, Kahle PJ, Springer W (2010). PINK1/Parkin-mediated mitophagy is dependent on VDAC1 and p62/SQSTM1. Nat Cell Biol.

[41] Wang H, Song P, Du L, Tian W, Yue W, Liu M, Li D, Wang B, Zhu Y, Cao C, Zhou J, Chen Q (2011). Parkin ubiquitinates Drp1 for proteasome-dependent degradation: implication of dysregulated mitochondrial dynamics in Parkinson disease. J Biol Chem.

[42] Sandebring A, Thomas KJ, Beilina A, van der Brug M, Cleland MM, Ahmad R, Miller DW, Zambrano I, Cowburn RF, Behbahani H, Cedazo-Minguez A, Cookson MR (2009). Mitochondrial alterations in PINK1 deficient cells are influenced by calcineurin-dependent dephosphorylation of dynamin-related protein 1. PLoS One.

[43] Jahani-Asl A, Slack RS (2007). The phosphorylation state of Drp1 determines cell fate. EMBO reports.

[44] Otera H, Ishihara N, Mihara K (2013). New insights into the function and regulation of mitochondrial fission. Biochim Biophys Acta.

[45] O'Flanagan CH, Morais VA, Wurst W, B De Strooper, O'Neill C (2015). The Parkinson's gene PINK1 regulates cell cycle progression and promotes cancer-associated phenotypes. Oncogene.

[46] Lee KS, Lu B (2014). Targeting PINK1 and MQC in brain tumors. Oncotarget.

[47] Li G, Zhou T, Liu L, Chen J, Zhao Z, Peng Y, Li P, Gao N (2013). Ezrin dephosphorylation/downregulation contributes to ursolic acid-mediated cell death in human leukemia cells. Blood Cancer J.

