# Supplementary material for: Polyphyllin I induces mitophagic and apoptotic cell death in human breast cancer cells by increasing mitochondrial PINK1 levels
Source: Oncotarget. 2017 Jan 2;8(6):10359–74. doi: 10.18632/oncotarget.14413 (PMC5354664; doi:10.18632/oncotarget.14413)
Supplement: Supplementary file 1 [file oncotarget-08-10359-s001.pdf]

Polyphyllin I induces mitophagic and apoptotic cell death in human breast cancer cells by increasing mitochondrial PINK1 levels

SUPPLEMENTARY FIGURES

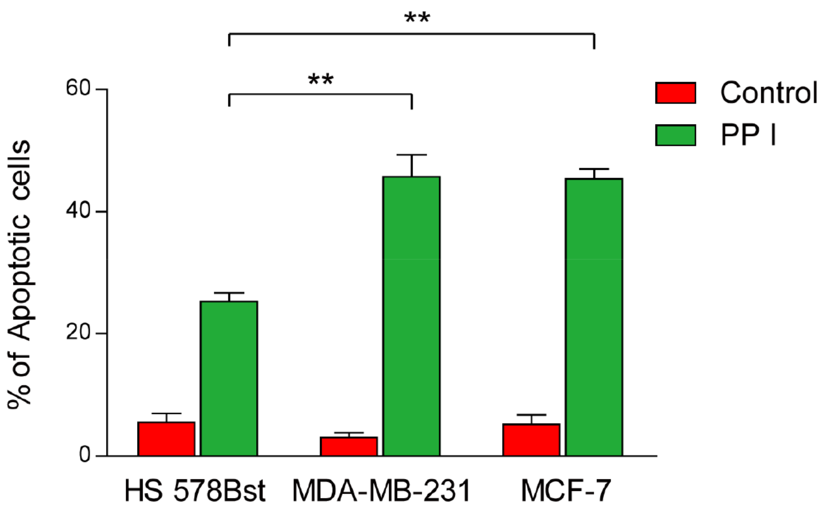

Supplementary Figure 1: Polyphyllin I induces apoptosis in Hs-578Bst, MDA-MB-231, and MCF-7 cells. Human mammary stromal cells (Hs-578Bst) and mammary cancer cells (MDA-MB-231, MCF-7) were treated with 8 μM PPI for 9 h, after which cells were stained with Annexin V-FITC/PI and apoptosis was detected by flow cytometry. Data are presented as mean ± SD (**$P < 0.01$ compared to Hs-578Bst cells treated with PPI).

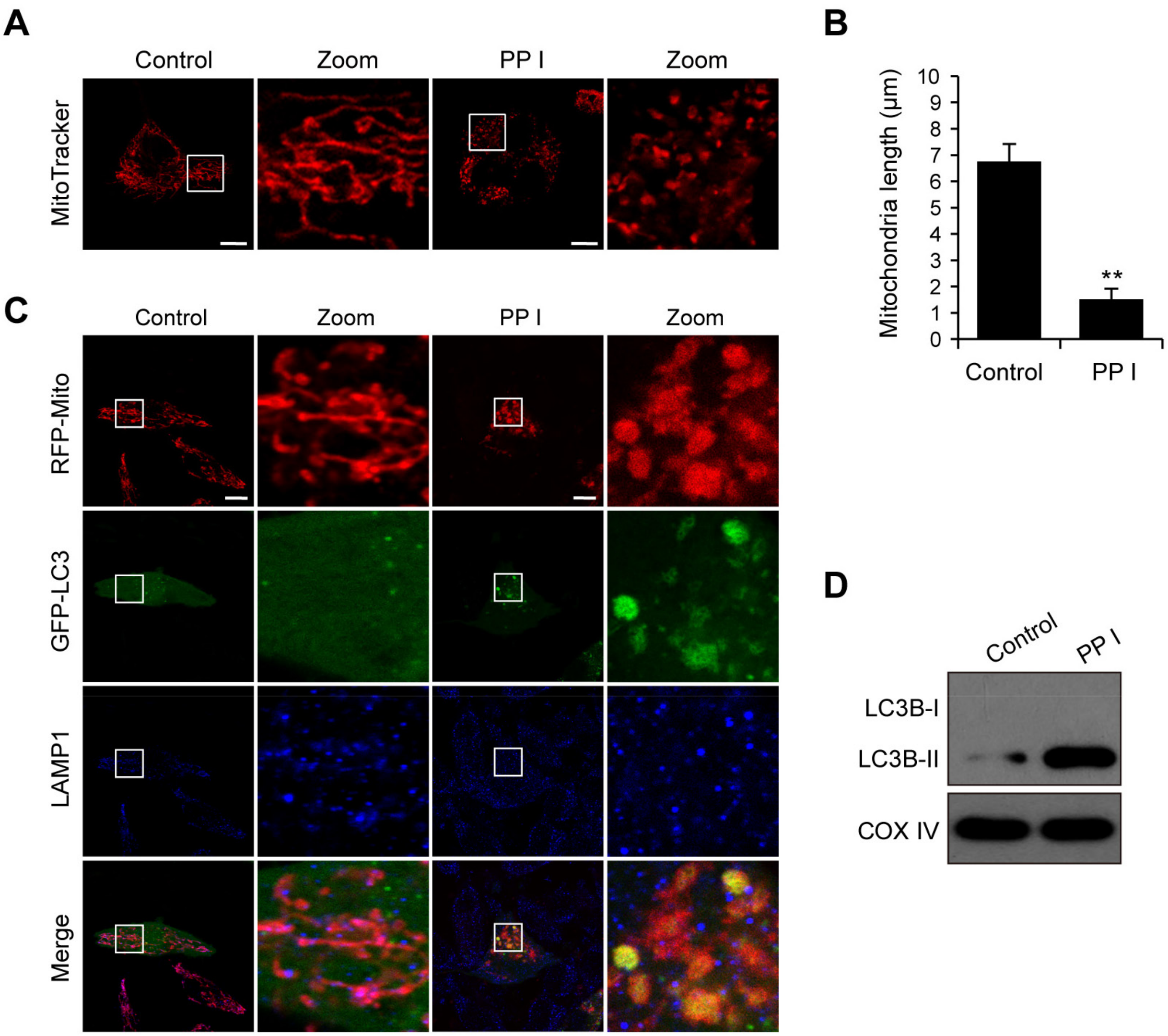

Supplementary Figure 2: Polyphyllin I induces mitochondrial fission and mitophagy in MCF-7 cells. A. MCF-7 cells were treated with or without 8 μM polyphyllin I for 9 h, and mitochondrial morphology was then examined using MitoTracker Red CMXRos staining and confocal microscopy. Scale bars: 10 μm. **B.** Average mitochondrial length was quantified for 30 cells in each experiment; 3 independent experiments are included ($^{**}P < 0.01$). **C.** Cells were cotransfected with RFP-mito and GFP-LC3 and then treated with 8 μM polyphyllin I for 9 h. LAMP1 (Alexa Fluor 647, blue) immunostaining was then examined using confocal microscopy. Scale bars: 10 μm. **D.** LC3 expression in mitochondrial fractions was determined by western blot in cells treated as described in A.

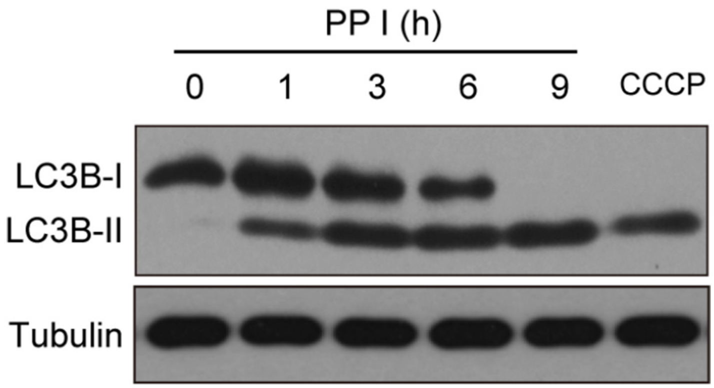

Supplementary Figure 3: LC3B expression after polyphyllin I treatment. MDA-MB-231 cells were treated with 8 μM PPI for different periods of time as indicated or with 20 μM CCCP for 9 h; LC3B expression was then examined in whole-cell lysates using western blot analysis.

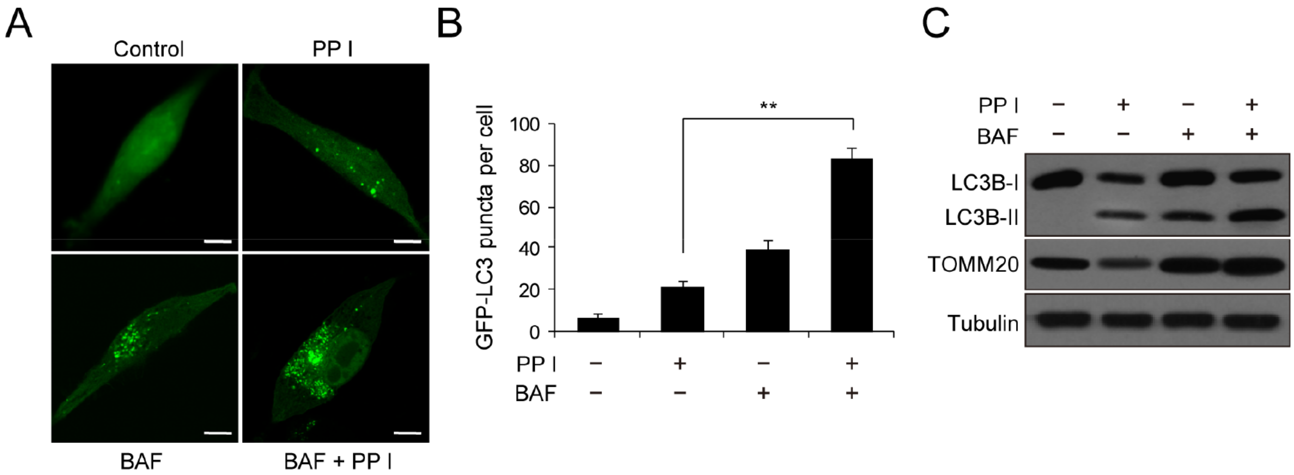

Supplementary Figure 4: Polyphyllin I induces autophagy. **A**. MDA-MB-231 cells were transfected with GFP-LC3 and exposed to 2 µM polyphyllin I in the presence or absence of 25 nM bafilomycin A_1 (BAF) for 9 h; GFP-LC3 puncta were then examined using confocal microscopy. Scale bars: 10 µm. **B**. Average numbers of GFP puncta per cell were quantified for 30 cells in each experiment; 3 independent experiments are included. Data are presented as mean ± SD (**$P < 0.01$ compared to cells treated with PPI). **C**. LC3B-I/LC3B-II and TOMM20 expression were analyzed by western blot analysis.

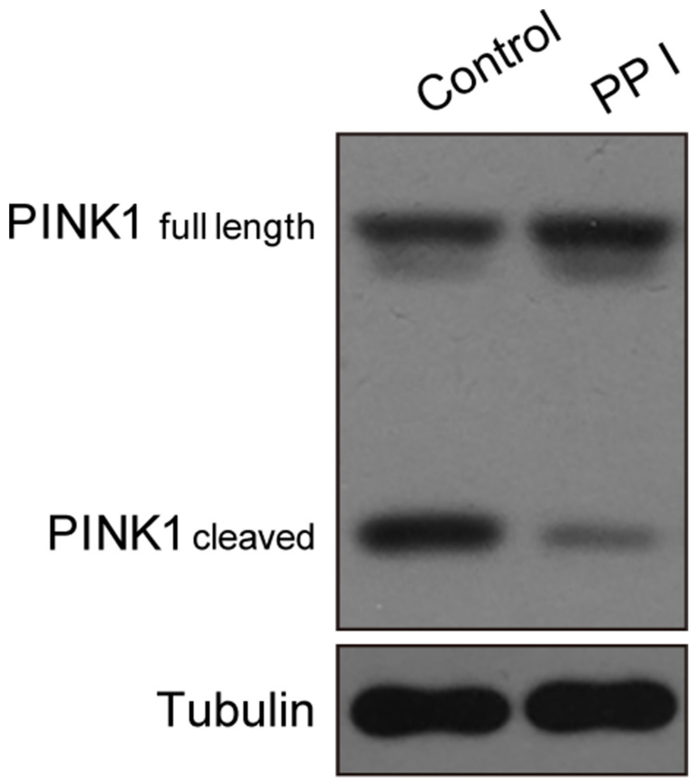

Supplementary Figure 5: Polyphyllin I blocks PINK1 cleavage in MCF-7 cells. MCF-7 cells were treated with or without 8 μM polyphyllin I for 6 h, and PINK1 expression was then analyzed by western blot.

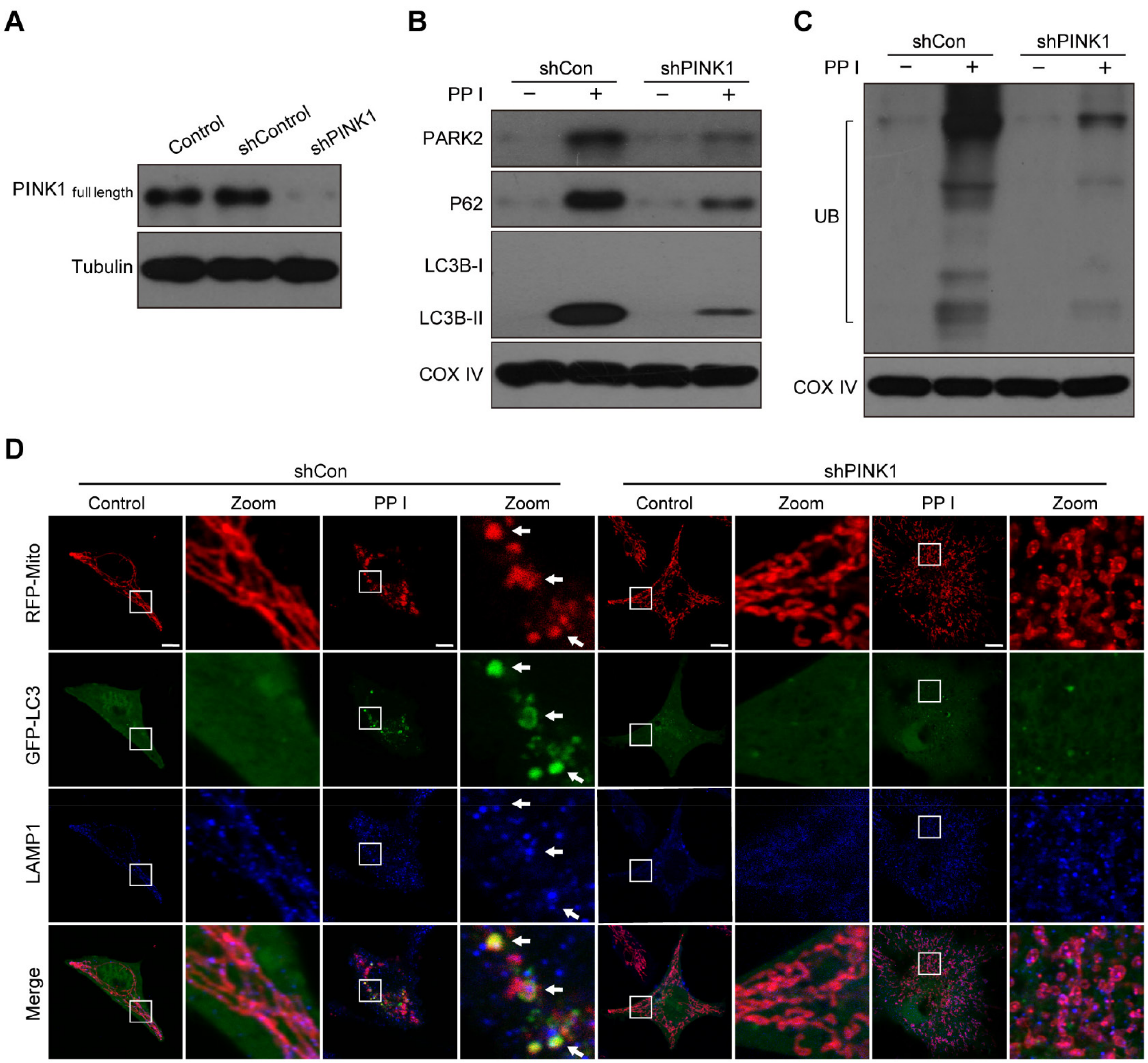

Supplementary Figure 6: PINK1 knockdown suppresses polyphyllin I-induced mitophagy in MCF-7 cells. A. MCF-7 cells stably expressing non-target shRNA (shCon) or PINK1 shRNA (shPINK1) were lysed and analyzed by western blot using the anti-PINK1 antibody. **B-C.** shCon and shPINK1 cells were treated with or without 8 μM polyphyllin I for 6 h; PARK2, P62, LC3, and ubiquitin (UB) expression were then examined in mitochondrial fractions by western blot. **D.** shCon and shPINK1 cells were cotransfected with RFP-mito and GFP-LC3 and then treated with 8 μM polyphyllin I for 6 h. LAMP1 (Alexa Fluor 647, blue) immunostaining was then examined using confocal microscopy. Scale bars: 10 μm.

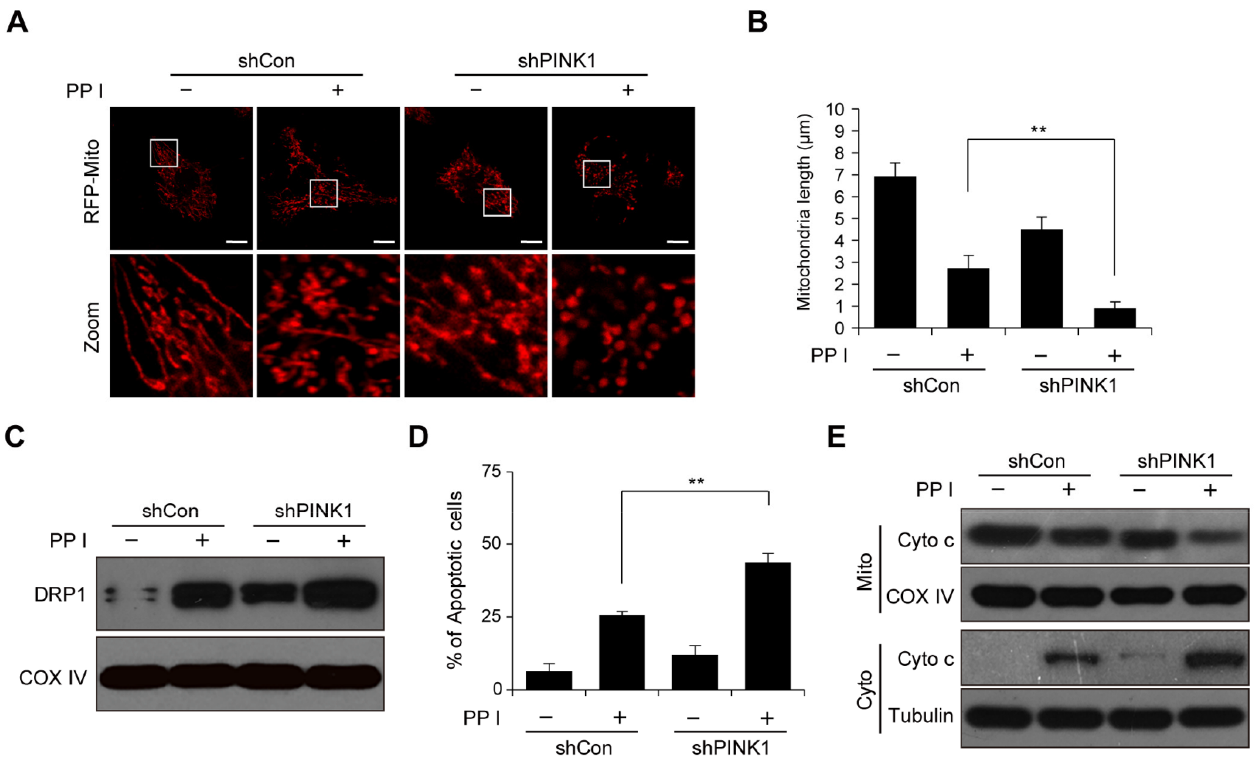

Supplementary Figure 7: PINK1 knockdown increases polyphyllin I-induced mitochondrial translocation of DRP1, fission, and apoptosis in MCF-7 cells. A. Cells stably expressing shCon or shPINK1 MCF-7 were transfected with RFP-mito and then treated with 8 µM polyphyllin I for 6 h. Mitochondria were examined using confocal microscopy. Scale bars: 10 µm. **B.** Average mitochondrial length was quantified for 30 cells in each experiment; 3 independent experiments are included ($^{**}P < 0.01$ compared to shCon cells treated with PPI). **C** and **E.** Mitochondrial (Mito) and cytosolic (Cyto) fractions were prepared and subjected to western blot analysis. **D.** Cells were treated with or without 8 µM polyphyllin I for 6 h, and apoptosis was then measured by flow cytometry. Data are presented as mean ± SD ($^{**}P < 0.01$ compared to shCon cells treated with PPI).
